# Factors Associated With Engaging in Regular Physical Activity Among Women Family Caregivers

**DOI:** 10.1093/geroni/igab046.1891

**Published:** 2021-12-17

**Authors:** Abiola Keller

**Affiliations:** Marquette University, Milwaukee, Wisconsin, United States

## Abstract

Regular physical activity is important for promoting the health of family caregivers. In this study, we used data from the 2015 and 2017 Behavioral Risk Factor Surveillance System Questionnaire-Caregiver module to examine factors associated with meeting physical activity guidelines among women caregivers. Meeting physical activity guidelines was defined as participating in 150 minutes (or vigorous equivalent minutes) of physical activity weekly. We used survey-weighted multivariate regression analyses to examine relationships between sociodemographic, caregiving, and health characteristics and meeting physical activity guidelines. All variables were entered into the model simultaneously. The Wald test was used to test the significance of interactions between race and ethnicity and other covariates. 50.7% of 10,542 women caregivers met physical activity guidelines. The amount of time spent caregiving each week was not associated with the odds of meeting guidelines. Caregivers in the paid workforce had decreased odds (OR=0.73, 95%CI [0.62-0.87]) of meeting guidelines. Compared to women caregiving for <6months, women caregiving for 6 months to 2 years had increased odds of meeting guidelines (OR =1.33, 95%CI [1.08-1.64]). Increasing education was associated with an increased odds of meeting guidelines, but being college educated had a more positive effect for Hispanic than white caregivers (pinteraction=0.03). Having children did not affect the odds of meeting guidelines for white caregivers, but for black caregivers having two or more children decreased the odds (pinteraction=0.03). Understanding how sociodemographic, caregiving, and health characteristics impact engagement in regular physical activity is critical to designing effective interventions and ultimately improving the health of caregivers.

